# Physiological, anthropometric and athletic performance adaptations from completing a 1-month pre-season period. A two-year longitudinal study in female collegiate soccer players

**DOI:** 10.3389/fspor.2024.1353129

**Published:** 2024-03-11

**Authors:** Andrew S. Perrotta, Brent D. Day, Camila J. Correa, Anika J. Scott, Jennifer Ramos, Elizabeth A. Gnatiuk, Darren E. R. Warburton

**Affiliations:** ^1^ Department of Kinesiology, Faculty of Human Kinetics, University of Windsor, Windsor, ON, Canada; ^2^ Department of Kinesiology, Centre for Human Performance and Health, Windsor, ON, Canada; ^3^ Department of Kinesiology, Langara College, Vancouver, BC, Canada; ^4^ School of Kinesiology, Faculty of Education, University of British Columbia, Vancouver, BC, Canada; ^5^ Experimental Medicine Program, Faculty of Medicine, University of British Columbia, Vancouver, BC, Canada

**Keywords:** sport science, exercise testing, athlete development, athlete monitoring, female athletes

## Abstract

**Introduction:**

Collegiate coaches and integrative support staff often utilize pre-season as a brief and intense training period to prepare athletes technically, tactically, and physiologically, to meet the demands of competition during a soccer season. This study sought to examine the dose-response from performing on-field soccer activities during a four-week pre-season period in female collegiate soccer players, and if the magnitude in response was associated with accumulated exercise stress.

**Methods:**

A total of twenty-seven healthy female soccer players training as part of a collegiate soccer program volunteered to participate in this two-year longitudinal study. Data collection commenced prior to the start of each pre-season period, at the beginning of August, and was completed at the beginning of September, when pre-season ended. Data collection periods were separated by a 31-day period. Indices of cardiovascular function, anthropometry, and athletic performance were examined during each data collection period. Internal and external measures of accumulated exercise stress were recorded using the Polar Team Pro® system.

**Results:**

When comparing the beginning to the end of pre-season, significant improvements were observed in body fat (%) [24.2 ± 6.0 “vs.” 23.3 ± 5.6, *p* = 0.001], heart rate variability (rMSSD) [51.8 ± 25.1 “vs.” 67.9 ± 34.6 ms, *p* = 0.002], resting heart (bpm) [73.8 ± 12.1 “vs.” 64.3 ± 8.8, *p* = 0.001] and cardiorespiratory performance (YoYo IRTL-1) [925.8 ± 272.8 “vs.” 1,062.6 ± 223.3 m, *p* = 0.001]. Significant reductions in musculoskeletal performance were observed through vertical jump height (cm) [24.9 ± 23.7, *p* = 0.04]. Change in the end of pre-season body weight (kg) was significantly associated with accumulated accelerations and decelerations [*r* ≥ 0.49, *p* = 0.01]. End of pre-season change in cardiorespiratory performance was significantly associated with both accumulated training load (au) and TRIMP (au) [*r* ≥ 0.63, *p* = 0.01].

**Discussion:**

In conclusion, performing a four-week pre-season period, involving only on-field training, can promote positive and significant adaptations in anthropometry, cardiovascular function, and athletic performance measures in female collegiate soccer players. The magnitudes of these adaptations were associated with both internal and external measures of accumulated exercise stress.

## 1 Introduction

The use of global positioning systems (GPS) that can quantify external work completed have revealed collegiate female soccer players cover significant amounts of distance over the course of a game, in addition to performing actions that require superior musculoskeletal performance (1). These demands are largely supported through elevated and sustained cardiorespiratory and musculoskeletal function that lead to substantial caloric expenditure (2). As such, practitioners within collegiate soccer routinely implement physical performance testing within the yearly training plan to examine adaptations in anthropometry, cardiorespiratory and musculoskeletal performance in response to exercise and competition (3). Integrative support staff have an assortment of efficient and non-invasive assessments at their disposal to examine measures of biological function that have shown to respond to exercise and support athletic performance. Resting cardiovascular measures such as heart rate variability (HRV) and resting heart rate (HR) have demonstrated significant associations with superior cardiorespiratory performance in female collegiate soccer players (4, 5). Furthermore, these measures have revealed their utility for examining the dose-response to exercise in collegiate soccer players (6), as well as their effectiveness in supporting integrative support staff in monitoring athletic development and fatigue over a season (7).

Adaptations in anthropometry in response to exercise and competition have been well examined in female collegiate soccer players (8). Practitioners habitually examine body composition over a season to better understand its adaptations to various stressors, as well as to predict an athlete's ability to execute tactical and technical actions on-field (9). The effect of body composition on athletic performance measures in female soccer players has recently been demonstrated by Stankoiv et al., where increases in fat free mass exhibited significant, and positive associations to musculoskeletal function when measured using vertical jump performance, agility, as well as running speed. Additionally, decreases in body fat (%) were observed to be significantly associated to enhanced cardiorespiratory performance (10). As such, strength and conditioning coaches place large significance towards prescribing exercise programs that are either designed to maintain or improve muscle mass depending on the training cycle within the yearly program. However, there remains a paucity of inquiry towards understanding the dose-response from exercise and competition in female soccer players when solely playing the sport and its impact on anthropometry. To date, the majority of research has focused on examining variations in body composition over singular or multiple seasons, all of which involve concurrent training methods, using both resistance training, endurance training and on-field soccer activities (11, 12). The utility in quantifying exercise stress elicited through soccer specific training alone and understanding its association with physiological and anthropometric adaptations would support coaches and integrative support staff in designing both on-field and off-field training programs.

A collegiate soccer season typically spans from late August until November. This brief schedule involves condensed match play where teams compete twice a week over a 72 h period and involves intensified on-field preparation throughout the week. The pre-season period for a female collegiate soccer program is often limited to 2–7 weeks depending on the collegiate sport regulating body. This period is viewed as a last chance opportunity for coaches to improve their team's technical, tactical, and physical capabilities before entering the competitive period, involving intensified training and significant alterations in physiological functioning (13). The primal focus on accumulating large training loads during this condensed and intensified training period can lead to increased injuries rates (14) and has displayed trivial evidence to support on-field success over a competitive season (15).

These concentrated training periods should be viewed with caution as either an opportunity to establish a foundation in physical fitness, or an opportunity to improve both physiological and athletic performance. As such, this investigation sought to examine the dose-response from performing soccer specific activities designed towards improving the technical and tactical performance of players as well as their physiological functioning during a four-week pre-season period in female collegiate soccer players. In conducting this study, we hypothesized the following: (1) significant alterations in anthropometry, cardiovascular function, and athletic performance measures would be observed after completing pre-season, and (2) accumulated exercise stress would be significantly correlated to improvements in anthropometry, cardiovascular function, and athletic performance measures.

## 2 Methods

### 2.1 Study design

This investigation was a two-year longitudinal study, examining the association between exercise stress and adaptations in physiological function and sport performance from completing a pre-season period in female collegiate soccer players. Data collection occurred at the start of August and finished at the beginning of September for each pre-season and was separated by a 31-day period. A total of three competitive matches were included during each pre-season and were performed on alternate weekends. On-field training sessions were 90-min in length and occurred Monday through Thursday. Training sessions were structured to begin with a warm-up and were followed by technical, tactical, and physiologically oriented soccer drills, concluding with a cool-down. Both inter-reliability and intra-reliability were controlled by using standardized examiners over the two-year period.

Participants followed their own nutrition, hydration, and sleep patterns throughout the investigation. Data collection was standardized for a given time of day, with participants arriving at a controlled laboratory setting between 09:00 and 11:00. All anthropometric, physiological, and athletic performance measures were examined together during each laboratory visit. Hydration and nutrition were standardized 24-hr prior to collection period, with all physical activity ceasing 48-hr prior to testing. Each data collection followed the order of; (1) resting cardiovascular function, (2) anthropometric analysis, (3) musculoskeletal function, (4) linear running speed, and (5) cardiorespiratory performance. All testing occurred in a controlled environment with ambient temperatures of 20.1°C (± 1.2°C) and a relative humidity of 30.4% (± 8.5%).

### 2.2 Participants

A total of twenty-seven (n = 27) healthy female soccer players training as part of a collegiate soccer program volunteered for this study. Over the two-year study period, the mean (± SD) participant age was 20.6 ± 1.2 year. Participant characteristics over the study period are displayed in Table 1. Each participant provided written informed consent to participate in the study and completed a health screening questionnaire prior to each pre-season period (16). Participants who were under the legal age (<19.0 year) at the time of the study had a parent or guardian provide their signature on the consent form. The current investigation was approved by and followed the recommendations of the Langara College Research Ethics Board and were in accordance with the ethical standards of use of human participants in research as outlined in the 1964 Declaration of Helsinki and its later amendments.

**Table 1:** Change in anthropometry over a preseason period.

	Beginning of preseason	End of preseason	% diff	*p* value	Effect size (95% CI)
Height (cm)	166.0 ± 7.0	166.0 ± 7.0	0.00%	1.000	0.00
Weight (kg)	57.3 ± 6.6	57.4 ± 6.6	0.01%	0.630	0.01 (± 0.04)
BF %	24.2 ± 6.0	23.3 ± 5.6	−3.70%	0.001	0.16 (± 0.09)
BMI (kg/m^2^)	20.7 ± 1.5	20.8 ± 1.5	0.17%	0.600	0.02 (± 0.08)

Values displayed as a mean (± SD). Values are as follows; BF, body fat %; BMI, body mass index.

### 2.3 Physiological assessments

Upon arrival for each data collection, a 10-min stabilization period was implemented whereby participants laid supine in a quiet room with their eyes closed before each cardiovascular assessment. Blood pressure (mmHg) and heart rate (bpm) were recorded using an automated blood pressure device (Welch Allyn Inc., New York, USA) utilizing an inflatable arm cuff positioned 3 cm above the antecubital space over the left brachial artery. Using indices of blood pressure and heart rate, stroke volume (mL·beat^−1^) was estimated using the Liljestrand and Zander (17) mathematical model and adapted to derive cardiac output (L·min^−1^) (18). Systematic vascular resistance (dynes⋅sec⋅cm^−5^) was calculated according to Ohm's law, whereby cardiac output and the resulting mean arterial pressure can derive resistance (19). Participants were assigned a POLAR Team Pro® chest strap heart rate monitor (Polar, Electro, Oy, Kempele, Finland) equipped to sample at 1,000 Hz. An ultra-short recording period of 60-sec was used to collect R-R intervals (ms) under spontaneous breathing while in the supine position. Data was then exported into Kubios HRV 3.0 (Biosignal Analysis and Medical Imagining Group at the Department of Applied Physics, University of Kuopio, Kuopio, Finland) for HRV analysis. The square root of the mean squared differences of successive R–R intervals (rMSSD), was selected for examining cardiac parasympathetic modulation based on its validity to represent cardiac vagal activity (20), its resilience against respiratory sinus arrhythmia during spontaneous breathing (21), and when not accounting for both menstrual cycle phase (22) and oral contraceptive use (23). A cubic spline interpolation method utilizing a third-degree polynomial that was pre-programmed into Kubios HRV 3.0, was used to interpret artifacts and ectopic beats that varied greater than 0.25 s when compared to the mean (24).

#### 2.3.1 Anthropometric assessments

Body weight and body composition were recorded without shoes or socks using a weight scale and bioelectrical impedance (Tanita BF-680® scale). Height (cm) was recorded using a portable stadiometer (Seca 217®) and was used in combination with body mass to calculate body mass index (kg/m^2^). Participant clothing was standardized for each assessment.

#### 2.3.2 Athletic performance measures

Musculoskeletal performance was examined using vertical ground reaction forces. Vertical jump height (cm) was estimated using a dual force plate system utilizing a 1 kHz operating frequency, a temporal resolution of (±0.25 N) and a mechanical range of (0–14 kN) (Hawkin Dynamics Inc. DE, USA). Participants placed their hands on their hips before descending to a knee angle of approximately 90° and held this position for three seconds before performing a vertical jump. Three attempts were completed with a 10-sec recovery period between jumps to promote recovery and to avoid muscular fatigue. The average of the three jumps was used for analysis (25). Running speed was measured at both 5 m and 10 m distances using photocell gates (Brower TC Timing Systems, UT, USA) and recorded as time (sec). Participants held a stationary position for three seconds before sprinting through each photocell. The best of three attempts, using a three-minute rest period between sprints, was used for analysis. Maximal cardiorespiratory performance was assessed using the YoYo Intermittent Recovery Test Level-1 (YoYo IRTL-1) (26). The last completed shuttle, and its corresponding distance (m) was recorded for analysis.

#### 2.3.3 Exercise stress

At the beginning of each training session and competitive match, participants were provided a Polar Team Pro® monitor equipped with a GPS unit (10 Hz) and heart rate monitor (1-sec intervals) for recording indices of exercise stress (27). Upon completion of the cool down from each activity, data was exported into the Polar Team Pro® cloud-based software. Accumulated exercise stress throughout each pre-season period was quantified using both internal and external measures of exercise stress. Internal measures were derived from exercise heart rate dynamics and were quantified as a training impulse (TRIMP) (28) and as a Polar training load (29). Total energy expenditure (kcal) was estimated from the Polar Pro software and was calculated using exercise heart rate dynamics and each participant's unique anthropometry, age, sex, height, weight, resting and maximum heart rate, as well as estimated VO2max as established from the YoYo IRTL-1. External measures recorded were the total distance covered (km) as well as the number (#) of sprints, accelerations and decelerations performed. Thresholds for detecting both sprints and accelerations were manually inputted into the Polar Team Pro system and were specified using the slowest 30 m sprint speed (15 km/h) and 5 m acceleration time (2.8 m.s^2^) recorded from the participants in the study.

#### 2.3.4 Statistical analysis

Data was analyzed using Microsoft® EXLSTAT 5.1. All data in text, tables and figures are displayed as means (± SD). Normality of data sets were examined using a Shapiro-Wilk's test for normality (*p* > 0.05) prior to analysis. A paired samples t-test was used to examine changes in physiological function and sport performance from the beginning to the end of pre-season. In addition, standardized mean differences were calculated to display the effect size using Cohen's *d*, and were accompanied with a 95% CI. Effect size values were interpreted as follows; small (*d *= 0.20), medium (*d* = 0.50) and large (*d* = 0.80) (30).

A Pearson correlation coefficient (r) was used to examine associations between indices of accumulated exercise stress and the change observed in physiological, anthropometric, and performance measures from the beginning to the end of pre-season. The following principles were applied for identifying the strength of correlation; *r* = 0.10–0.29 small, *r* = 0.30–0.49 moderate, *r* > 0.50 large (30). Significance was declared using a probability of *p* < 0.05.

## 3 Results

Data is presented as means (± SD) unless otherwise stated. Accumulated internal exercise stress was recorded as the following: calories = 14,792.7 ± 3,725.8 (kcal), training load = 3,062.5 ± 884.3 (AU), TRIMP = 3,368.7 ± 892.9 (AU). Accumulated external exercise stress was recorded as the following: total distance covered = 106,751.3 ± 33,858.0 (km), sprints = 1,425.9 ± 469.8 (#), accelerations = 103.2 ± 49.2, and decelerations = 131.0 ± 69.0 (#). Changes in anthropometry are displayed in Table 1. Alterations in resting cardiovascular function are shown in Table 2. Changes in athletic performance measures are presented in Table 3. Individual correlation matrices displaying the association between accumulated exercise stress and: (1) change in cardiovascular function (Table 4), change in anthropometry (Table 5), change in athletic performance (Table 6) are provided.

**Table 2:** Change in cardiovascular function over a preseason period.

	Beginning of preseason	End of preseason	% diff	*p* value	Effect size (95% CI)
RHR (bpm)	73.8 ± 12.1	64.3 ± 8.8	−v12.9	0.001	−0.83 (± 0.46)
rMSSD (ms)	51.8 ± 25.1	67.9 ± 34.6	30.9	0.002	−0.52 (± 0.31)
R-R (ms)	832.7 ± 125.4	951.4 ± 137.8	14.2	0.001	−0.83 (± 0.46)
Q (L·min^−1^)	5.3 ± 1.2	4.3 ± 0.66	−18.8	0.001	−0.92 (± 0.51)
SV (mL·beat^−1^)	72.3 ± 10.2	67.4 ± 7.5	−6.3	0.045	−0.49 (± 0.48)
Systolic (mmHg)	110.2 ± 7.0	109.5 ± 6.2	−0.63	0.563	−0.10 (± 0.06)
Diastolic (mmHg)	66.3 ± 5.5	68.2 ± 5.5	2.8	0.116	0.33 (± 0.42)
PP (mmHg)	43.9 ± 6.5	41.1 ± 4.3	−5.8	0.050	−0.45 (± 0.45)
MAP (mmHg)	77.9 ± 16.4	78.9 ± 16.6	1.2	0.323	0.06 (± 0.12)
SVR (dynes·sec·cm^−5^)	1,273.5 ± 297.3	1,544.0 ± 235.9	21.2	0.001	0.91 (± 0.51)

Values displayed as a mean (± SD). Values are as follows; RHR, resting heart rate; rMSSD, root mean square of successive differences in R-R intervals; R-R, beat-to-beat intervals; Q, cardiac output; SV, stroke volume; PP, pulse pressure; MAP, mean arterial pressure; SVR, systemic vascular resistance.

**Table 3:** Change in athletic performance measures over a preseason period.

	Beginning of preseason	End of preseason	% diff	*p* value	Effect size (95% CI)
5 m running speed (s)	1.17 ± 0.09	1.20 ± 0.07	2.9	0.090	0.44 (± 0.51)
10 m running speed (s)	2.00 ± 0.08	2.02 ± 0.07	1.2	0.300	0.31 (± 0.60)
Vertical jump height (cm)	24.9 ± 3.6	23.70 ± 3.3	−4.9	0.040	−0.35 (± 0.33)
YoYo IRTL-1 (m)	925.8 ± 272.8	1,062.6 ± 223.3	8.3	0.001	0.56 (± 0.24)

Values displayed as a mean (± SD).

**Table 4:** Correlation matrix. Associations between accumulated exercise stress and change in cardiovascular function.

	Calories	Training load	TRIMP	Total distance	Sprints	Acc	Dec	RHR	rMSSD	R-R	Q	SV	Systolic	Diastolic	PP	MAP	SVR
Calories (kcal)	**–**																
Training load (AU)	0.90**	**–**															
TRIMP (AU)	0.92**	0.96**	**–**														
Total distance (km)	0.64**	0.77**	0.78**	**–**													
Sprints (#)	0.50**	0.66**	0.64**	0.71**	**–**												
Acc (#)	−0.20	−0.16	−0.18	−0.04	0.29**	**–**											
Dec (#)	−0.03	0.05	0.03	0.21	0.50	0.87**	**–**										
RHR (bpm)	−0.09	−0.16	−0.12	0.00	0.02	0.17	0.18	**–**									
rMSSD (ms)	0.19	0.01	0.15	0.03	−0.13	−0.37	−0.29	−0.21	**–**								
R-R (ms)	0.08	0.12	0.06	−0.09	−0.09	−0.13	−0.13	−0.90**	0.14	**–**							
Q (L·min^−1^)	−0.13	−0.19	−0.16	−0.06	−0.11	−0.04	−0.06	0.80**	−0.17	−0.69**	**–**						
SV (mL·beat^−1^)	−0.14	−0.19	−0.15	−0.12	−0.19	−0.20	−0.25	0.37	0.00	−0.28	0.84**	**–**					
Systolic (mmHg)	0.53**	0.56**	0.59**	0.47*	0.39*	−0.18	−0.20	0.12	−0.03	−0.17	0.21	0.20	**–**				
Diastolic (mmHg)	0.45*	0.50**	0.49**	0.40*	0.41*	0.08	0.11	−0.27	−0.02	0.15	−0.64**	−0.79**	0.45*	**–**			
PP (mmHg)	0.10	0.08	0.12	0.09	0.00	−0.25	−0.30	0.37	−0.01	−0.31	0.80**	0.92**	0.56**	−0.49**	**–**		
MAP (mmHg)	0.55**	0.60**	0.61**	0.49**	0.47*	−0.01	0.00	−0.16	−0.03	0.05	−0.40*	−0.51**	0.74**	0.93**	−0.14	**–**	
SVR (dynes·sec·cm^−5^)	0.25	0.32	0.28	0.20	0.22	0.11	0.16	−0.68**	−0.03	0.63	−0.91**	−0.86**	−0.01	0.78**	−0.73**	0.59**	**–**

Values are provided as a Pearson correlation coefficient. Acc, acceleration; Dec, deceleration; TRIMP, training impulse; RHR, resting heart rate; rMSSD, root mean square of successive beat-to-beat intervals; R-R, beat to beat cardiac intervals; Q, cardiac output; SV, stroke volume; PP, pulse pressure; MAP, mean arterial pressure; SVR, systemic vascular resistance.

* *p* < 0.05.

** *p* < 0. 01.

**Table 5:** Correlation matrix. Associations between accumulated exercise stress and change in anthropometry.

	Calories	Training load	TRIMP	Total distance	Sprints	Acc	Dec	Body weight	BMI	Body fat
Calories (kcal)	**–**									
Training load (AU)	0.90**	**–**								
TRIMP (AU)	0.92**	0.96**	**–**							
Total distance (km)	0.64**	0.77**	0.78**	**–**						
Sprints (#)	0.50**	0.66**	0.64**	0.71**	**–**					
Acc (#)	−0.20	−0.16	−0.18	−0.04	0.29	**–**				
Dec (#)	−0.03	0.05	0.03	0.21	0.50**	0.87**	**–**			
Body weight (kg)	−0.18	−0.13	−0.18	−0.02	0.20	0.49**	0.52**	**–**		
BMI (kg/m^2^)	−0.18	−0.17	−0.20	−0.07	0.08	0.43*	0.47*	0.97**	**–**	
Body Fat (%)	0.04	0.19	0.06	0.07	0.08	−0.10	0.04	0.37	0.44*	**–**

Values are provided as a Pearson correlation coefficient. TRIMP, training impulse; Acc, acceleration; Dec, deceleration; BMI, body mass index.

* p < 0.05.

** p < 0.01.

**Table 6:** Correlation matrix. Associations between accumulated exercise stress and changes in athletic performance.

	Calories	Training load	TRIMP	Total distance	Sprints	Acc	Dec	5 m running speed	10 m running speed	Vertical jump height	YoYo ITRL-1
Calories (kcal)	**–**										
Training load (AU)	0.81**	**–**									
TRIMP (AU)	0.85**	0.90**	**–**								
Total distance (km)	0.37	0.61**	0.63**	**–**							
Sprints (#)	0.01	0.32	0.25	0.49*	**–**						
Acc (#)	−0.28	−0.28	−0.32	−0.07	0.39	**–**					
Dec (#)	−0.23	−0.16	−0.19	0.12	0.53**	0.88**	**–**				
5 m running speed (sec)	0.03	−0.12	−0.12	−0.17	−0.14	0.10	−0.06	**–**			
10 m running speed (sec)	0.01	−0.16	−0.15	−0.22	−0.20	0.16	−0.04	0.99**	**–**		
Vertical jump height (cm)	−0.41*	−0.22	−0.24	−0.10	0.17	0.04	0.16	−0.25	−0.28	**–**	
YoYo IRTL−1 (m)	0.71**	0.76**	0.63**	0.43*	0.15	−0.48*	−0.26	−0.14	−0.20	−0.02	**–**

Values are provided as a Pearson correlation coefficient. TRIMP, training impulse; Acc, acceleration; Dec, deceleration; BMI, body mass index.

* *p* < 0.05.

** *p* < 0.01.

## 4 Discussion

This investigation sought to examine the dose-response in female collegiate soccer players when performing on-field soccer activities during a four-week pre-season period. The primary observations revealed from this study were significant improvements in cardiac parasympathetic activity with concomitant improvements in cardiorespiratory performance in addition to significant reductions in both body fat percentage and musculoskeletal performance. The magnitude of change in anthropometric, cardiovascular, and athletic performance measures were significantly associated with accumulated exercise stress over the pre-season period.

Strength and conditioning coaches place a strong focus towards developing a healthy body composition in soccer players. Increases in fat free mass can lead to superior musculoskeletal performance as a result of increased contractile units, while enhanced body composition can reduce cardiovascular strain whereby improving cardiorespiratory performance (10). Our hypothesis was confirmed with a meaningful decrease in body fat % of −3.7%, a change beyond that of the typical error of 2.5% (31). Although we failed to observe a significant association between change in body fat % and accumulated exercise stress, we did record a significant association between increases in body weight and both accelerations and decelerations, suggesting change in fat free mass was associate with GPS metrics. During soccer competition, accelerations and decelerations can inflict unique physiological and mechanical loading (32), whereby greater metabolic processes are required during acceleration (33), while a larger mechanical load is experienced during deceleration (34). Nevertheless, both actions elicit significant muscular damage that can provoke intracellular signaling processes required for muscle remodeling (35). The significant increase in fat free mass observed in the current study may have been the result of sufficient intramuscular signaling coupled with adequate nutritional practices to support muscle remodeling.

Female collegiate soccer players who possess superior cardiorespiratory and musculoskeletal performance typically display enhanced on-field technical and tactical activities during competition (1). Practitioners and coaches often work together to design on-field training sessions to achieve the desired technical, tactical, and physiological oriented demands of their sport (36). Ideally, performing the sport itself to improve cardiorespiratory and musculoskeletal performance would negate the need for additional conditioning sessions. The reduction in additive training sessions would support physiological recovery and enhance the potential for improved competitive performance (37). The current study demonstrated a significant improvement (8.3%) in cardiorespiratory performance beyond that of the typical error (38). This increase was significantly associated with both internal and external indices of exercise stress. However, greater associations with internal measures were identified. This observation can be expected, as exercise heart rate dynamics are a reflection of cardiovascular stress, and a direct indicator for potential improvement in cardiorespiratory performance (39). This evidence supports integrative support staff in monitoring heart rate derived training loads during on-field training as this stimulus was shown to independently, and sufficiently, improve fitness levels. Although improvements in cardiorespiratory performance were observed, we noted decrements in musculoskeletal function. Vertical jump performance was significantly reduced (−4.9%) beyond that of the typical error of 3.0% (40). Although not statistically significant (*p* = 0.09), 5 m running speed was also reduced. Inverse associations between musculoskeletal performance and exercise stress were evident, however only caloric expenditure demonstrated a significant association with vertical jump performance. The time course of neuromuscular function and its effect of jump performance has shown to be impaired for up to five days post competition (41). This may be the result of either prolonged (> 48 h) muscle damage or alterations in neuromuscular excitation from training and competition (42). In the current study, end of pre-season assessments were completed 48 h after the cessation of all exercise, suggesting prolonged fatigue may have overshadowed any neuromuscular adaptations developed from pre-season. We recommend future studies employ multiple testing periods after exiting a pre-season period to allow for fatigue to dissipate to identify true changes in musculoskeletal performance. Moreover, this evidence suggests coaches consider tapering during the end of pre-season, prior to entering a competitive season, to facilitate recovery and allow for training adaptations to supersede accumulative fatigue.

The importance of cardiovascular function in female collegiate soccer players has most recently been demonstrated by Perrotta et al., where superior cardiorespiratory and musculoskeletal performance were observed in athletes with greater cardiac function. Our hypothesis was confirmed as we observed significant improvements in cardiovascular function as displayed through a −12.9% decrease in resting heart rate and a 30.9% increase in heart rate variability (rMSSD). The mechanisms for improved resting cardiac vagal activity from endurance exercise are complex and not fully understood. Yamamato et al., demonstrated endurance training can lead to enhanced cardiac vagal modulation, whereby directly decreasing cardiac tone (43). The participants in the current study began each pre-season after performing minimal training and competition in the off-season. Plasma volume has shown to rapidly expand in response to performing exercise at an intensity or volume that is uncustomary (44, 45). Although blood volume was not examined in the current study, we hypothesize that participants likely developed hypervolemia in response to the significant increase in exercise stress accumulated during pre-season. Exercise induced hypervolemia has lead to improvements in cardiac vagal modulation, a result of increased afferent activity of the baroreflex loop in response to blood volume expansion (46). Furthermore, an expansion in plasma volume may have enhanced cardiac function, and may in part explain the significant increase in maximal cardiorespiratory performance (47).

The current study is not without its limitations. Anthropometry was examined using bioelectrical impedance which is limited to estimating changes in whole body composition. The use of dual x-ray absorptiometry (DEXA) where segmental volume can be analyzed, would provide greater insight into the locational changes in body composition and their associations to accumulated exercise stress. Additionally, indirect methods were used to examine cardiovascular function. Although significant decrements in stroke volume, cardiac output, and systemic vascular resistance were observed, these changes were likely the result of a decrease in both heart rate and pulse pressure, and their use in the mathematical equation for estimating cardiac function (17, 18) and vascular resistance (19).

## 5 Practical applications

In practical terms for soccer coaches, it's essential to recognize the documented benefits of incorporating resistance training and cardiorespiratory exercises to support the musculoskeletal and cardiorespiratory demands of competition in female collegiate soccer players (1, 2). However, striking the right balance between each form of exercise alongside soccer-specific training demands is a common challenge. During preseason training, coaches often face the decision to focus primarily on physical fitness enhancement or on refining players’ technical and tactical skills. Trying to excel in all areas simultaneously can lead to excessive psychophysiological stress on players (13), potentially causing non-functional overreaching. Therefore, a collaborative approach between coaches and practitioners is crucial in designing training sessions that address both technical/tactical aspects as well as elicit adequate exercise stress to either maintain or improve physiological functioning. Integrating small-sided games that encourage repeated and rapid changes of direction, featuring significant accelerations and decelerations, as well as larger heart rate-derived training loads is an effective strategy to enhance players’ technical and physiological capacities simultaneously (48). This approach, as supported by results of the current study, can positively impact body composition and cardiorespiratory performance. Consequently, it may eliminate the necessity for additional resistance training sessions, promoting athletes’ recovery and overall development before the regular season begins.

## 6 Conclusion

Performing a four-week pre-season period, involving only technical, tactical and physiological oriented soccer training, can promote positive and significant adaptations in anthropometry, cardiovascular function, and athletic performance measures in female collegiate soccer players. The magnitudes of these adaptations are associated with both internal and external measures of accumulated exercise stress.

## Data Availability

The original contributions presented in the study are included in the article/Supplementary Material, further inquiries can be directed to the corresponding author.

## References

[1] Jagim AR Murphy J Schaefer AQ Askow AT Luedke JA Erickson JL Match demands of women’s collegiate soccer. Sports. (2020) 8(6):87. 10.3390/sports8060087 32545603 PMC7353669

[2] McFadden BA Walker AJ Bozzini BN Sanders DJ Arent SM. Comparison of internal and external training loads in male and female collegiate soccer players during practices vs. Games. J Strength Cond Res. (2020) 34(4):969–74. 10.1519/JSC.0000000000003485 31972824

[3] Ishida A Bazyler CD Sayers AL Stone MH Gentles JA. Evidence and application of athlete monitoring programs in national collegiate athletic association women’s soccer: a narrative review. J Strength Cond Res. (2022) 44(3):33–45. 10.1519/SSC.0000000000000670

[4] Perrotta AS Correa CJ Khan AD Day BD Warburton DER Ramos J. Resting cardiovascular function and athletic performance in female soccer players. Sci Sports. (2024) 39(1):112–6. 10.1016/j.scispo.2022.11.006

[5] Flatt AA Esco MR Nakamura FY Plews DJ. Interpreting daily heart rate variability changes in collegiate female soccer players. J Sports Med Phys Fitness. (2017) 57(6):907–15. 10.23736/S0022-4707.16.06322-2 26997322

[6] Flatt AA Esco MR Nakamura FY. Individual heart rate variability responses to preseason training in high level female soccer players. J Strength Cond Res. (2017) 31(2):531–8. 10.1519/JSC.0000000000001482 27227794

[7] Mascarin RB De Andrade VL Barbieri RA Loures JP Kalva-Filho CA Papoti M. Dynamics of recovery of physiological parameters after a small-sided game in women soccer players. Front Physiol. (2018) 9:887. 10.3389/fphys.2018.00887 30050459 PMC6050376

[8] Peart AN Nicks CR Mangum M Tyo BM. Evaluation of seasonal changes in fitness, anthropometrics, and body composition in collegiate division II female soccer players. J Strength Cond Res. (2018) 32(7):2010–7. 10.1519/JSC.0000000000002578 29570578

[9] McFadden BA Walker AJ Arent MA Bozzini BN Sanders DJ Cintineo HP Biomarkers correlate with body composition and performance changes throughout the season in women’s division I collegiate soccer players. Front Sports Act Living. (2020) 2:74. 10.3389/fspor.2020.00074 33345065 PMC7739727

[10] Stanković M Čaprić I Đorđević D Đorđević S Preljević A Koničanin A Relationship between body composition and specific motor abilities according to position in elite female soccer players. Int J Environ Res Public Health. (2023) 20(2):1327. 10.3390/ijerph20021327 36674082 PMC9858742

[11] Lesinski M Prieske O Helm N Granacher U. Effects of soccer training on anthropometry, body composition, and physical fitness during a soccer season in female elite young athletes: a prospective cohort study. Front Physiol. (2017) 8:1093. 10.3389/fphys.2017.01093 29375392 PMC5770736

[12] Katona A Riewe C Bruzina A Ollberding NJ Ankrom M Divine J Body composition changes over multiple academic years in female collegiate soccer players. J Funct Morphol Kinesiol (2020) 5(4):72. 10.3390/jfmk5040072 33467287 PMC7739407

[13] Botelho R Abad CCC Spadari RC Winckler C Garcia MC Guerra RLF. Psychophysiological stress markers during preseason among elite female soccer players. J Strength Cond Res. (2022) 36(6):1648–54. 10.1519/JSC.0000000000003702 35622110

[14] Chandran A Morris SN Boltz AJ Robison HJ Collins CL. Epidemiology of injuries in national collegiate athletic association women’s soccer: 2014–2015 through 2018–2019. J Athl Train. (2021) 56(7):651–8. 10.4085/1062-6050-372-20 34280264 PMC8293894

[15] Coppalle S Rave G Ben Abderrahman A Ali A Salhi I Zouita S Relationship of pre-season training load with in-season biochemical markers, injuries and performance in professional soccer players. Front Physiol. (2019) 10:426414. 10.3389/fphys.2019.00409 PMC6474299 31031638

[16] Warburton DE Jamnik V Bredin SS Shephard RJ Gledhill N. The 2019 physical activity readiness questionnaire for everyone (PAR-Q+) and electronic physical activity readiness medical examination (ePARmed-X+). Health Fit J Can. (2018) 11(4):80–3.

[17] Liljestrand G Zander E. Vergleichende bestimmungen des minutenvolumens des herzens beim menschen mittels der stickoxydulmethode und durch blutdruckmessung. Zeitschrift für die Gesamte Experimentelle Medizin. (1928) 59(1):105–22. 10.1007/BF02608853

[18] Sun J Reisner A Saeed M Mark R. Estimating cardiac output from arterial blood pressurewaveforms: a critical evaluation using the MIMIC II database. Computers in Cardiology, 2005; Lyon, France (2005).

[19] Hill LK Sollers Iii JJ Thayer JF. Resistance reconstructed estimation of total peripheral resistance from computationally derived cardiac output - biomed 2013. Biomed Sci Instrum. (2013) 49:216–23. 23686203 PMC4444041

[20] Variability HR. Standards of measurement, physiological interpretation, and clinical use. Task force of the European Society of Cardiology and the North American society of pacing and electrophysiology. Circulation. (1996) 93:1043–65. 10.1161/01.CIR.93.5.1043 8598068

[21] Penttilä J Helminen A Jartti T Kuusela T Huikuri HV Tulppo MP Time domain, geometrical and frequency domain analysis of cardiac vagal outflow: effects of various respiratory patterns. Clin Physiol. (2001) 21(3):365–76. 10.1046/j.1365-2281.2001.00337.x 11380537

[22] Pestana ER Mostarda CT Silva-Filho AC Salvador EP de Carvalho WRG. Effect of different phases of menstrual cycle in heart rate variability of physically active women. Sport Sci Health. (2018) 14(2):297–303. 10.1007/s11332-018-0426-5

[23] Teixeira AL Ramos PS Vianna LC Ricardo DR. Heart rate variability across the menstrual cycle in young women taking oral contraceptives. Psychophysiology. (2015) 52(11):1451–5. 10.1111/psyp.12510 26332575

[24] Alcantara JMA Plaza-Florido A Amaro-Gahete FJ Acosta FM Migueles JH Molina-Garcia P Impact of using different levels of threshold-based artefact correction on the quantification of heart rate variability in three independent human cohorts. J Clin Med. (2020) 9(2):325. 10.3390/jcm9020325 31979367 PMC7074236

[25] Mercer RAJ Russell JL McGuigan LC Coutts AJ Strack DS McLean BD. Finding the signal in the noise—interday reliability and seasonal sensitivity of 84 countermovement jump variables in professional basketball players. J Strength Cond Res. (2023) 37(2):394–402. 36696261 10.1519/JSC.0000000000004182

[26] Krustrup P Mohr M Amstrup T Rysgaard T Johansen J Steensberg A The yo-yo intermittent recovery test: physiological response, reliability, and validity. Med Sci Sports Exercise. (2003) 35(4):697–705. 10.1249/01.MSS.0000058441.94520.32 12673156

[27] Akyildiz Z Yildiz M Clemente FM. The reliability and accuracy of polar team pro GPS units. Proc Inst Mech Eng P: J Sports Eng Technol. (2022) 236(2):83–9.

[28] Banister EW Morton RH Fitz-Clarke J. Dose/response effects of exercise modeled from training: physical and biochemical measures. Ann Physiol Anthropol. (1992) 11(3):345–56. 10.2114/ahs1983.11.345 1642735

[29] Nissilä J Kinnunen H. Heart rate based training load and recovery time estimation. Polar Electro Oy. (2008) 1:1–4.

[30] Cohen J. Statistical power analysis for the behavioral sciences. In: Hillsdale NJ, editors. 2nd edn. Lawrence Erlbaum Associates Inc (1988).

[31] Vasold KL Parks AC Phelan DML Pontifex MB Pivarnik JM. Reliability and validity of commercially available low-cost bioelectrical impedance analysis. Int J Sport Nutr Exerc Metab. (2019) 29(4):406–10. 10.1123/ijsnem.2018-0283 30507268

[32] Verheul J Nedergaard NJ Pogson M Lisboa P Gregson W Vanrenterghem J Biomechanical loading during running: can a two mass-spring-damper model be used to evaluate ground reaction forces for high-intensity tasks? Sports Biomech. (2021) 20(5):571–82. 10.1080/14763141.2019.1584238 31033415

[33] Hader K Mendez-Villanueva A Palazzi D Ahmaidi S Buchheit M. Metabolic power requirement of change of direction speed in young soccer players: not all is what it seems. PLoS One. (2016) 11(3):e0149839. 10.1371/journal.pone.0149839 26930649 PMC4773143

[34] Dalen T Jørgen I Gertjan E Havard HG Ulrik W. Player load, acceleration, and deceleration during forty-five competitive matches of elite soccer. J Strength Cond Res. (2016) 30(2):351–9. 10.1519/JSC.0000000000001063 26057190

[35] Gastin PB Hunkin SL Fahrner B Robertson S. Deceleration, acceleration, and impacts are strong contributors to muscle damage in professional Australian football. J Strength Cond Res. (2019) 33(12):3374–83. 10.1519/JSC.0000000000003023 30694964

[36] Perrotta AS Taunton JE Koehle MS White MD Warburton DER. Monitoring the prescribed and experienced heart rate-derived training loads in elite field hockey players. J Strength Cond Res. (2019) 33(5):1394–9. 10.1519/JSC.0000000000002474 29420388

[37] Fessi MS Zarrouk N Di Salvo V Filetti C Barker AR Moalla W. Effects of tapering on physical match activities in professional soccer players. J Sports Sci. (2016) 34(24):2189–94. 10.1080/02640414.2016.1171891 27065344

[38] Grgic J Oppici L Mikulic P Bangsbo J Krustrup P Pedisic Z. Test–retest reliability of the yo-yo test: a systematic review. Sports Med. (2019) 49:1547–57. 10.1007/s40279-019-01143-4 31270753

[39] Foster C Florhaug JA Franklin J Gottschall L Hrovatin LA Parker S A new approach to monitoring exercise training. J Strength Cond Res. (2001) 15(1):109–15. 11708692

[40] Thomas C Dos’Santos T Comfort P Jones PA. Between-Session reliability of common strength- and power-related measures in adolescent athletes. Sports (Basel). (2017) 5(1):15. 10.3390/sports5010015 29910375 PMC5969001

[41] West DJ Cook CJ Stokes KA Atkinson P Drawer S Bracken RM Profiling the time-course changes in neuromuscular function and muscle damage over two consecutive tournament stages in elite rugby sevens players. J Sci Med Sport. (2014) 17(6):688–92. 10.1016/j.jsams.2013.11.003 24332752

[42] Byrne C Twist C Eston R. Neuromuscular function after exercise-induced muscle damage: theoretical and applied implications. Sports Med. (2004) 34(1):49–69. 10.2165/00007256-200434010-00005 14715039

[43] Yamamoto K Miyachi M Saitoh T Yoshioka A Onodera S. Effects of endurance training on resting and post-exercise cardiac autonomic control. Med Sci Sports Exerc. (2001) 33(9):1496–502. 10.1097/00005768-200109000-00012 11528338

[44] Buchheit M Laursen P Al Haddad H Ahmaidi S. Exercise-induced plasma volume expansion and post-exercise parasympathetic reactivation. Eur J Appl Physiol. (2009) 105(3):471–81. 10.1007/s00421-008-0925-1 19009307

[45] Convertino VA. Blood volume: its adaptation to endurance training. Med Sci Sports Exercise. (1991) 23(12):1338–48. 10.1249/00005768-199112000-00004 1798375

[46] Billman GE Dickey D Teoh K Stone H. Effects of central venous blood volume shifts on arterial baroreflex control of heart rate. Am J Physiol. (1981) 241(4):H571–H5. 10.1152/ajpheart.1981.241.4.H571 7315981

[47] Gledhill N Warburton D Jamnik V. Haemoglobin, blood volume, cardiac function, and aerobic power. Can J Appl Physiol. (1999) 24(1):54–65. 10.1139/h99-006 9916181

[48] Gantois P Piqueras-Sanchiz F Cid MJFA Pino-Ortega J Castillo D Nakamura FY. The effects of different small-sided games configurations on heart rate, rating of perceived exertion, and running demands in professional soccer players. Eur J Sport Sci. (2023) 23(7):1214–22. 10.1080/17461391.2022.2092427 35723596

